# The ZEB1/miR-200c feedback loop regulates invasion via actin interacting proteins MYLK and TKS5

**DOI:** 10.18632/oncotarget.4807

**Published:** 2015-08-20

**Authors:** Vignesh Sundararajan, Nicolas Gengenbacher, Marc P. Stemmler, Julia A. Kleemann, Thomas Brabletz, Simone Brabletz

**Affiliations:** ^1^ Department of Visceral Surgery, University Medical Center Freiburg, Freiburg, Germany; ^2^ Spemann Graduate School of Biology and Medicine (SGBM), Albert-Ludwigs-University Freiburg, Freiburg, Germany; ^3^ Faculty of Biology, Albert-Ludwigs-University Freiburg, Freiburg, Germany; ^4^ Division of Vascular Oncology and Metastasis, German Cancer Research Center (DKFZ-ZMBH Alliance), Heidelberg, Germany; ^5^ Department of Experimental Medicine I, Nikolaus-Fiebiger-Center for Molecular Medicine, University Erlangen-Nürnberg, Erlangen, Germany

**Keywords:** epithelial to mesenchymal transition (EMT), ZEB1, miR-200, MYLK (MLCK), invadopodia

## Abstract

Epithelial to mesenchymal transition (EMT) is a developmental process which is aberrantly activated during cancer invasion and metastasis. Elevated expression of EMT-inducers like ZEB1 enables tumor cells to detach from the primary tumor and invade into the surrounding tissue. The main antagonist of ZEB1 in controlling EMT is the microRNA-200 family that is reciprocally linked to ZEB1 in a double negative feedback loop. Here, we further elucidate how the ZEB1/miR-200 feedback loop controls invasion of tumor cells. The process of EMT is attended by major changes in the actin cytoskeleton. Via *in silico* screening of genes encoding for actin interacting proteins, we identified two novel targets of miR-200c - TKS5 and MYLK (MLCK). Co-expression of both genes with ZEB1 was observed in several cancer cell lines as well as in breast cancer patients and correlated with low miR-200c levels. Depletion of TKS5 or MYLK in breast cancer cells reduced their invasive potential and their ability to form invadopodia. Whereas TKS5 is known to be a major component, we could identify MYLK as a novel player in invadopodia formation. In summary, TKS5 and MYLK represent two mediators of invasive behavior of cancer cells that are regulated by the ZEB1/miR-200 feedback loop.

## INTRODUCTION

The major cause of cancer related deaths is not the primary lesion, but distant metastases at different sites of the body. Metastasis can be triggered by aberrant activation of an epithelial to mesenchymal transition (EMT), a reversible program that allows partial or complete transition between an epithelial and a mesenchymal phenotype. During EMT, polarized epithelial cells that are immobile and tightly embedded in the epithelial cell layer, gain mesenchymal properties that enable the cells to breakdown the basement membrane, invade into the surrounding tissues and migrate to distant sites. EMT is activated by key signaling pathways, including the TGFβ and FGF pathway and converging in the stimulation of EMT activators, a group of transcription factors repressing epithelial gene expression. These include members of the snail family, of the bHLH family and of the ZFH family (ZEB1 and ZEB2) [1].

EMT-mediated tumor progression and invasion is also regulated by microRNAs. The miR-200 family inhibits EMT and stabilizes the epithelial phenotype. Two major targets of miR-200 are the EMT inducers ZEB1 and ZEB2. Reciprocally, the ZEB proteins are strong transcriptional repressors of all miR-200 family members (miR-141, -200a, b, c and -429), resulting in the double negative ZEB/miR-200 feedback loop [2–7]. This feedback loop is deregulated in several human cancers including breast, pancreatic, prostate and colon [8], whereby high ZEB1 and consequently low miR-200 levels are crucial already for the first step of metastasis, the invasion into the surrounding tissues [9, 10]. Invasion of cancer cells is linked to the formation of protrusive structures such as invadopodia. These dynamic structures are actin rich membrane protrusions, which display proteolytic activity on the extracellular matrix [11]. Invadopodia comprise a central actin-rich core surrounded by a group of adhesion, membrane remodeling, scaffolding, and matrix degradation proteins [12, 13]. Although most mechanistic studies regarding invadopodia formation are conducted *in vitro* in cell culture experiments, there is growing evidence for the importance of invadopodia *in vivo* in cancer metastasis formation [14–17].

To better dissect the broad effect of the ZEB1/miR-200 feedback loop in cancer cell invasion, we used an *in silico* screening approach together with expression data from our breast cancer EMT/MET-cell line model [9], to find relevant genes besides ZEB1, that are specifically inhibited by miR-200 to prevent cell invasion. Using this strategy, we found two novel miR-200 target genes, TKS5 (SH3PXD2A) and MYLK (MLCK). Whereas TKS5 is already known to be involved in invadopodia formation, we further identified MYLK as a new player in invadopodia formation that is essential for the invasion of breast cancer cells.

## RESULTS

### The ZEB1/miR-200c feedback loop regulates actin interacting genes

**Figure 1:**
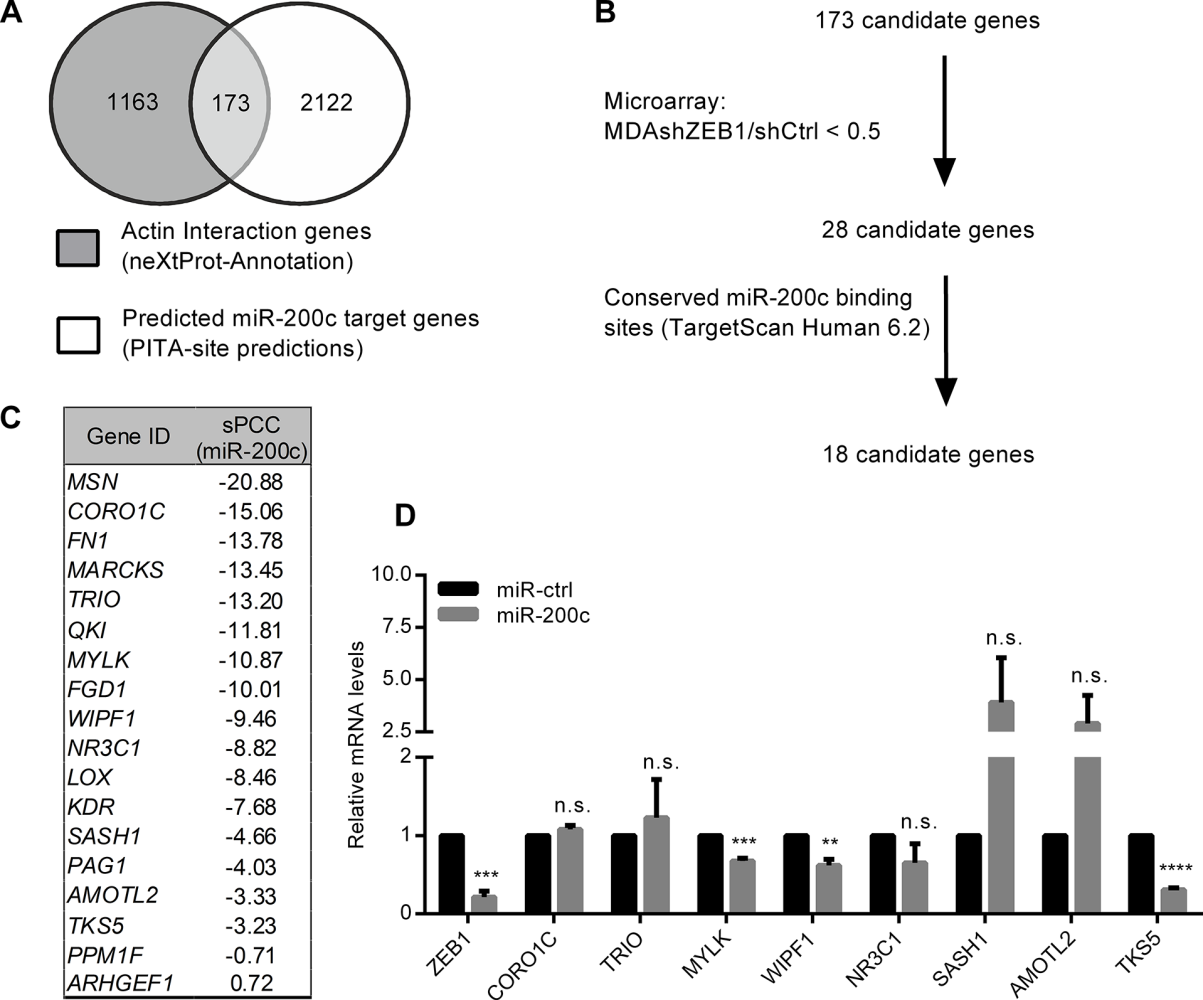
Identification of potential cell invasion target genes of miR-200c **A.** *In silico* screening of genes containing the term “actin interaction” in the functional annotation of *nextprot* database was merged to miR-200c targets predicted using the PITA target prediction program, resulting in 173 candidates. **B.** 18 of these 173 potential target genes harbor at least one miR-200c binding site that is conserved in vertebrates (TargetScanHuman6.2) and exhibit an expression pattern of <0.5 in the microarray analysis of MDA-MB-231 shZEB1/shCtrl. **C.** miRConnectL expression correlation of the 18 target genes with miR-200c (sPCC=summed Pearson correlation coefficient). **D.** Quantitative RT-PCR showing changes in expression of 8 candidate genes after transient overexpression of miR-200c in MDA-MB-231 in comparison to control miR (miR-ctrl). β-Actin was used for normalization.

To identify ZEB1/miR-200c target genes, that directly contribute to cell invasion by cytoskeletal re-organization, we extracted a set of 1163 actin interacting genes from the *nextprot* database and merged these with a second set of 2122 predicted miR-200c target genes from the *PITA site predictions* database (Figure 1A). We then compared the resulting 173 *in silico* candidates with expression array data of the mesenchymal/undifferentiated mammary cancer cell line MDA-MB-231 (shCtrl) in comparison to ZEB1 knockdown cells (shZEB1) [9]. We excluded those genes, whose expression was not differentially regulated after manipulation of the ZEB1 expression (Figure 1B). The remaining 28 candidate genes were further narrowed down by excluding genes that lacked conserved miR-200c binding sites predicted by *TargetScanHuman6.2* database. Correlation of the remaining 18 candidate genes' expression profiles with expression levels of miR-200c via *MiRConnect* [18] revealed the expected negative correlation for all genes, with the exception of ARHGEF1, as indicated by *summed pearson correlation coefficients* (sPPCs) (Figure 1C). Among the resulting 17 candidates, 9 genes (MSN, FN1, MARCKS, QKI, FGD1, LOX, KDR, PAG1 and PPM1F) have already been described as miR-200 target genes [19–26]. To validate the remaining candidates, we measured mRNA levels in MDA-MB-231 cells after transient overexpression of miR-200c. Similar to ZEB1, that was used as positive control, three candidate genes: MYLK, WIPF1 and TKS5 were significantly downregulated in miR-200c overexpressing cells (Figure 1D). During the course of our work WIPF1 was identified and characterized as a miR-200 target [27]. Therefore we excluded WIPF1 from further investigations.

### MYLK and TKS5 are direct targets of miR-200c

TKS5 (Tyrosine kinase substrate with five SH3 domains - SH3PXD2A) is essential for invadopodia formation in cancer cells. It acts as a SRC dependent scaffolding protein which recruits different effector proteins, including Cortactin and ADAM metalloproteases to initiate actin polymerization and extracellular matrix degradation [28, 29]. Myosin light chain kinase (MYLK) is a protein kinase, whose main known function is the phosphorylation of myosin light chain (MLC2) at Thr18 and Ser19 that is a prerequisite for the formation of contractile actomyosin-filaments. The MYLK gene encodes two different catalytic protein isoforms. Short MYLK is mainly expressed in smooth muscle tissue, whereas the long isoform dominates in non-muscle tissue [30]. This non-muscle specific isoform was shown to directly interact with Cortactin that is also a crucial factor for invadopodia formation [31, 32].

**Figure 2:**
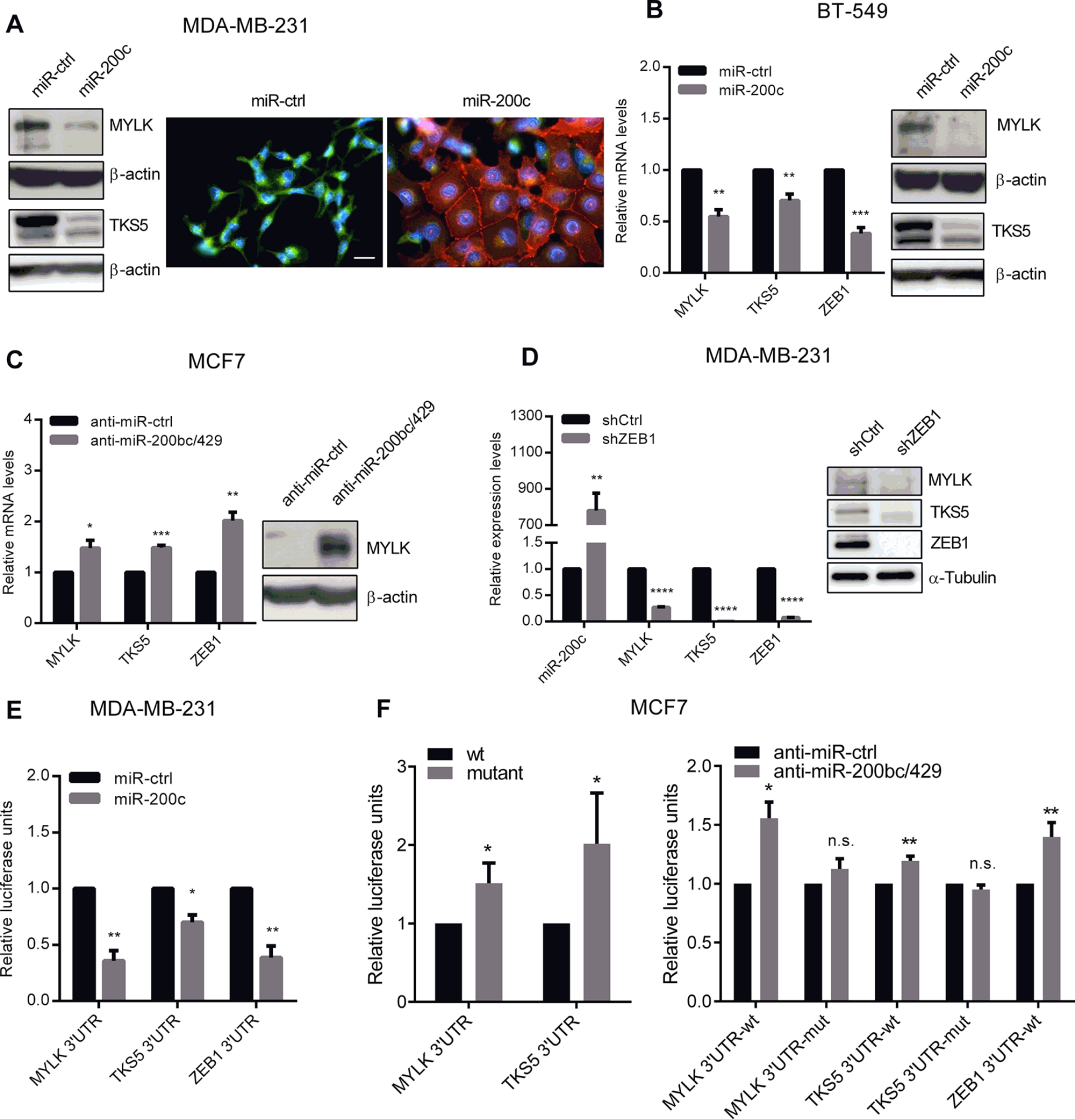
miR-200c overexpression leads to downregulation of MYLK and TKS5 **A.** Immunoblots (left) showing expression levels of MYLK and TKS5 after transient overexpression of miR-200c in MDA-MB-231. β-Actin was used as loading control. Immunofluorescence images (right) showing that transient overexpression of miR-200c in MDA-MB-231 significantly reduced TKS5 expression and increased E-cadherin expression. Scale bar, 20 μm. **B.** Quantitative RT-PCR (left) and immunoblots (right) showing expression levels of MYLK, TKS5 and ZEB1 after transient overexpression of miR-200c in BT-549. β-Actin was used for normalization in qPCRs and as loading control in immunoblots. **C.** Gene expression levels of MYLK, TKS5 and ZEB1, measured by qRT-PCR in MCF7 upon transient inhibition of the miR-200c subgroup (miR-200b, miR-200c and miR-429). Upregulation of MYLK on protein level was confirmed by immunoblot analysis (right). β-Actin was used as loading control. **D.** Gene expression levels of miR-200c, MYLK, TKS5 and ZEB1 in MDA-MB-231 cells with stable short hairpin RNA (shRNA) mediated knockdown of ZEB1 (shZEB1) versus control (shCtrl) showing downregulation of MYLK and TKS5 upon miR-200c activation. A respective immunoblot is given using Tubulin as loading control. **E.** Luciferase reporter assay of MYLK- and TKS5- 3′UTR showing reduced activity upon transient miR-200c overexpression in MDA-MB-231 cells compared to control miRNAs. The ZEB1- 3′UTR reporter was included as a positive control. **F.** Mutated versions of the MYLK- and TKS5- 3′UTR reporters allow higher relative luciferase activity in MCF7 cells, in comparison to the wt constructs (left). After inhibition of the miR-200c subgroup using antagomiRs only the wt, but not the mutated reporters were significantly upregulated (right). The ZEB1- 3′UTR reporter was included as a positive control.

To validate MYLK and TKS5 as direct miR-200c target genes, we first analyzed endogenous expression patterns in epithelial luminal type cell lines T47D and MCF7 with high miR-200 expression levels versus more mesenchymal basal type breast cancer cell lines Hs 578T and MDA-MB-231 exhibiting only very low miR-200 levels. In correlation to ZEB1, the high molecular weight, non-muscle isoform of MYLK, as well as TKS5 were highly expressed in basal type cell lines compared to the luminal types (Supplementary Figure S1). Transient overexpression of miR-200c in basal cell lines MDA-MB-231 and BT-549 significantly reduced MYLK and TKS5 mRNA- and protein levels (Figure 1D, 2A and 2B). Of note, as published previously [3, 33], the miR-200c overexpressing MDA MB 231 underwent an obvious EMT resulting in clustered cell growth with E-Cadherin positive adherence junctions and a cobble stone like epithelial morphology (Figure 2A). Conversely, selective inhibition of the miR-200c subgroup in the luminal cell line MCF7 resulted in upregulation of MYLK and TKS5 mRNA levels. MYLK was also upregulated on protein level (Figure 2C), whereas in spite of mRNA upregulation, TKS5 protein amounts remained below the detection limit. In order to confirm the contribution of ZEB1, we analyzed the expression of MYLK and TKS5 in MDA-MB-231 after ZEB1 knockdown in comparison to control cells. Upon stable ZEB1 knockdown, miR-200c levels were elevated, whereas the expression of MYLK and TKS5 was reduced (Figure 2D). These results indicate that expression of MYLK and TKS5 in breast cancer cell lines is controlled by the ZEB1/miR-200c feedback loop.

Accordingly, we found putative binding sites of miR-200c in the 3′-untranslated region (3′UTR) of MYLK and TKS5 that were highly conserved during evolution (Supplementary Figure S2). We generated luciferase reporter vectors, harboring the 3′UTRs containing these highly conserved binding sequences. Compared to control miRNA, overexpression of miR-200c in MDA-MB-231 cells significantly reduced luciferase activity of MYLK- and TKS5-3′UTR reporters (Figure 2E). Since ZEB1 is a well characterized validated miR-200 target [2–4, 7], the ZEB1-3′UTR reporter was used as a positive control [3]. We then mutated the putative miR-200c binding sites and found that the mutated reporter constructs exhibited higher activity in the differentiated, miR-200 high, breast cancer cell line MCF7 than the wt reporters. Accordingly, the selective inhibition of miR-200c subgroup in these cells led to a significant increase in luciferase activity only of wt MYLK-, TKS5- and ZEB1-3′UTR reporter constructs. Using the mutated MYLK- or TKS5-reporter constructs, no significant change in luciferase activity was observed (Figure 2F). These results confirm that MYLK and TKS5 are direct targets of miR-200c.

### Expression of MYLK and TKS5 correlates to ZEB1 and inversely correlates to miR-200c in cancer cells lines and breast cancer patients

**Figure 3:**
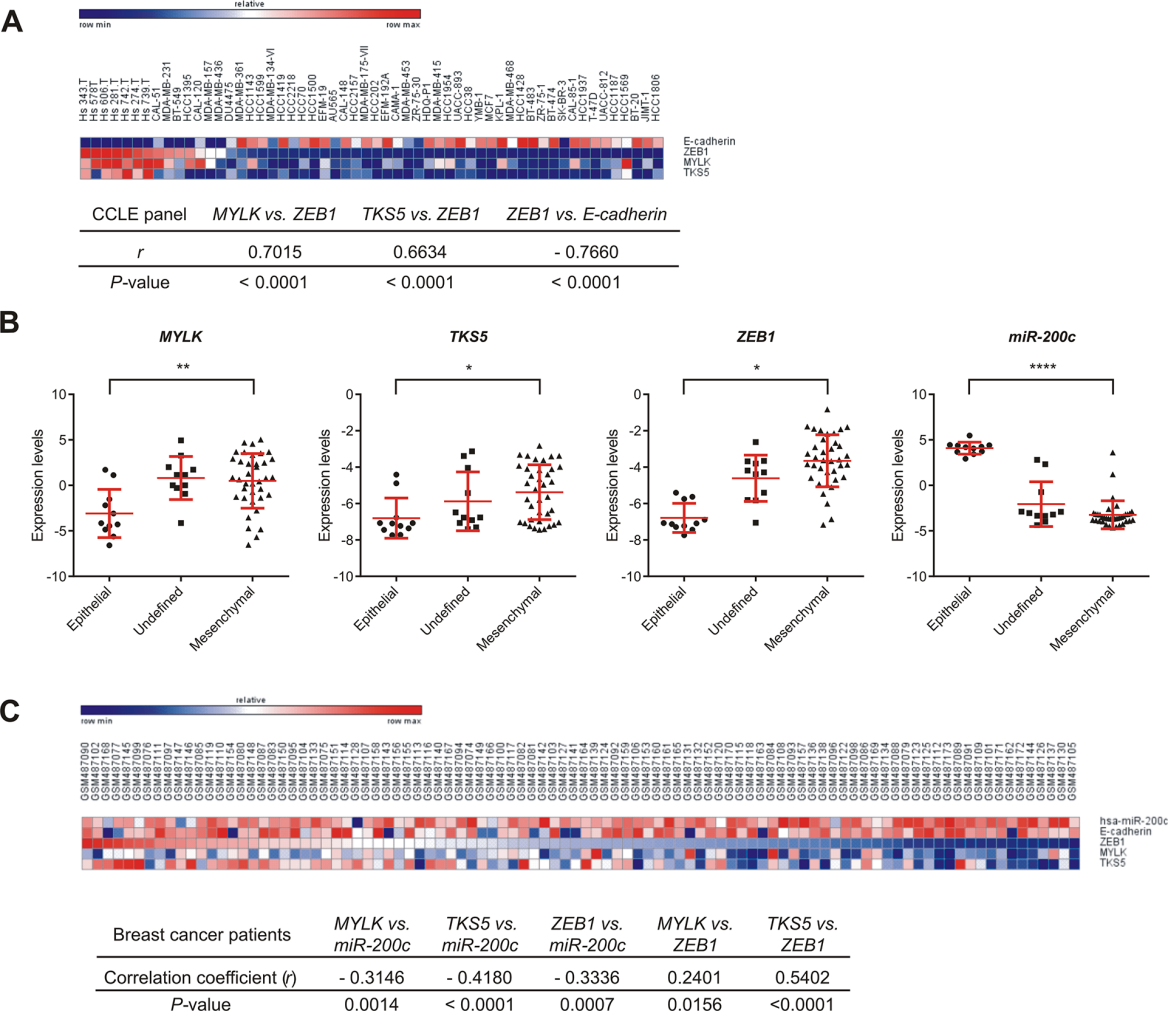
Expression correlation of MYLK, TKS5 and ZEB1 with miR-200c and/or E-cadherin in cancer cell lines and samples from breast cancer patients **A.** Heat map and expression correlation analysis of MYLK, TKS5, ZEB1 and E-cadherin shown for 56 breast cancer cell lines of the CCLE panel (GSE36133). Pearson correlation coefficients (r) and *P*-values (two-tailed) computed are shown below. **B.** NCI60 cell lines were classified as epithelial, undefined and mesenchymal clusters (according to ref [6]). Expression levels of MYLK, TKS5 and ZEB1 in these groups show significant enrichment in the mesenchymal cluster, whereas miR-200c is strongly enriched in the epithelial cluster. **C.** Heat map showing expression levels of miR200c, E-cadherin, ZEB1, MYLK and TKS5 for 101-breast cancer patients (GSE19783). Pearson correlation coefficients (r) and *P*-values (two-tailed) computed are shown below.

To assess the regulation of MYLK and TKS5, we performed correlation analyses in several datasets derived from human cancer cell lines. Analysis of gene expression 56 breast cancer cell lines from the Cancer Cell Line Encyclopedia (CCLE) panel [34] also showed high positive correlations between MYLK/TKS5 and ZEB1 (Figure 3A). Additionally, in the NCI60 panel, which comprises cell lines from nine different cancer entities, we observed clear inverse correlations of expression between miR-200c and MYLK/TKS5 (Supplementary Figure S3). Also, significant positive correlations were observed between ZEB1 and MYLK/TKS5. Moreover, when the NCI60 panel was classified in an epithelial, a mesenchymal or an undefined group [6], MYLK, TKS5 and ZEB1 expression was significantly enriched in the mesenchymal group. In contrast, miR-200c expression was high in the epithelial group (Figure 3B). Taken together, these data underline the relevance of ZEB1/miR-200c regulating MYLK and TKS5 expression in a wide panel of cancer cell lines. To further support our findings, we performed a similar correlation analysis using expression data from breast cancer patients. We analyzed a publicly available mRNA and miRNA microarray dataset from 101 breast cancer patients [35] and observed significant inverse correlations of miR-200c with its target genes MYLK, TKS5 and ZEB1 (Figure 3C). Also, significant positive correlations were observed between ZEB1 and MYLK/TKS5. These data suggest a contribution of the ZEB1/miR-200c feedback loop to the regulation of MYLK and TKS5 in breast cancer patients.

### MYLK and TKS5 affect invasion of breast cancer cells

**Figure 4:**
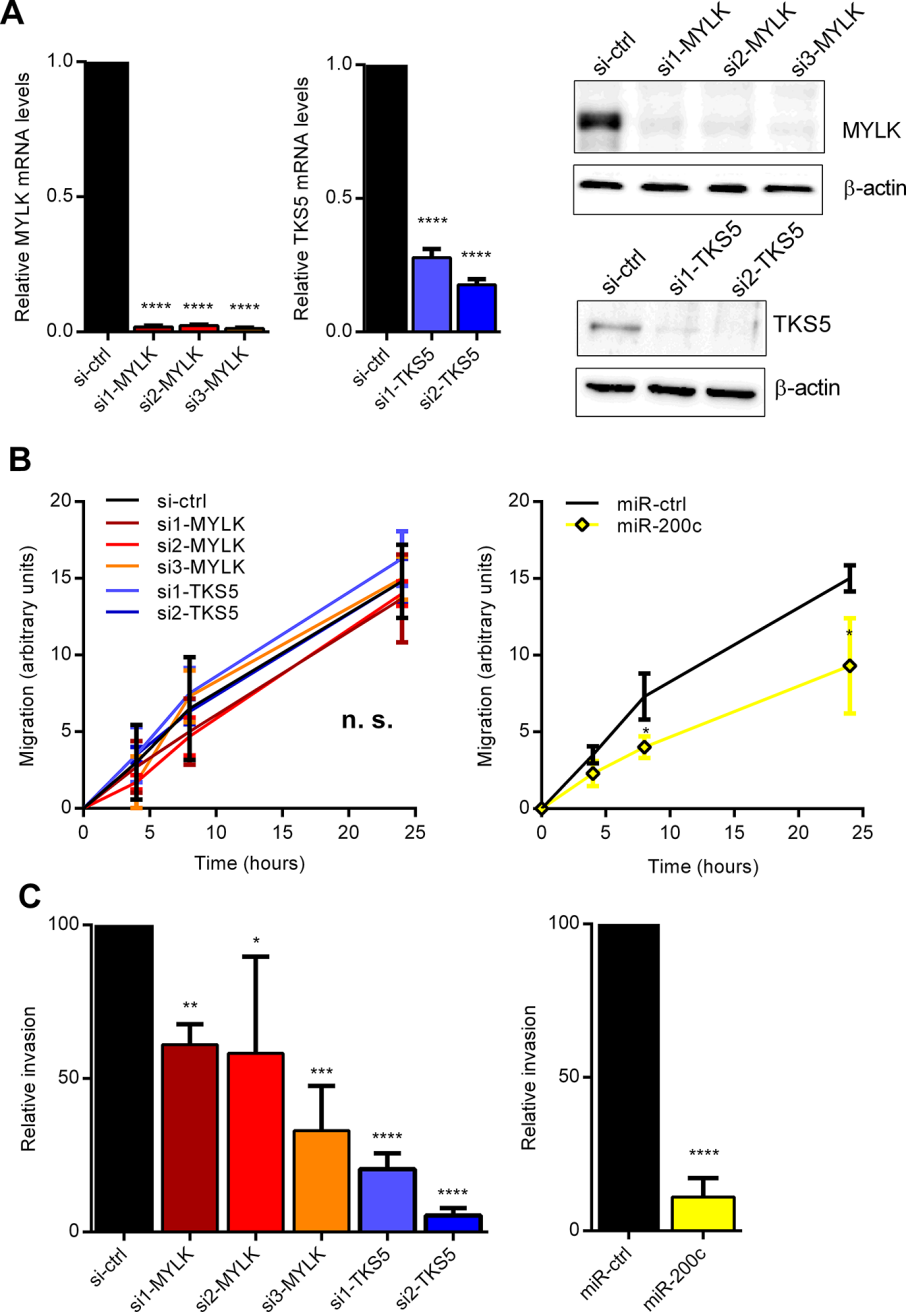
Inhibition of MYLK and TKS5 attenuates cancer cell invasion **A.** Quantitative RT-PCR and immunoblot analysis showing diminished expressing levels after transient siRNA-mediated knockdown of MYLK (left) and TKS5 (right) in MDA-MB-231. β-Actin was used as normalization gene and immunoblot loading control. **B.** Wounding assay for MDA-MB-231, transfected with si-MYLK, si-TKS5 or miR-200c. Quantification of three biological replicates shows a significant reduction in migration relative to control cells only after miR-200c overexpression, but not after si-MYLK or si-TKS5 treatment. Representative images are shown in Supplementary Figure S4A. **C.** Cultrex invasion assays for MDA-MB-231, transfected with si-MYLK, si-TKS5 or miR-200c. Quantification of three biological replicates shows a significant reduction in invasion relative to control cells after si-MYLK or si-TKS5 treatment as well as after miR-200c overexpression.

We and others have previously shown that miR-200c strongly reduces cancer cell migration and invasion, highlighting its role in tumor progression [3, 5, 36]. Therefore, we investigated whether individual knockdown of MYLK or TKS5 can phenocopy the effects of miR-200c overexpression on cancer cell migration and invasion. Selective silencing of MYLK and TKS5 via transient siRNA-mediated knockdown in MDA-MB-231 cells (Figure 4A), had no effect in real-time migration assays, whereas miR-200c overexpression significantly reduced the migration capacity in comparison to control miRNA transfected cells (Figure 4B and Supplementary Figure S4A, S4B). However, we could show that knockdown of either MYLK or TKS5 significantly impaired cancer cell invasion, thereby phenocopying at least partly the effect of miR-200c overexpression (Figure 4C and Supplementary Figure S4C). These data indicate that regulation of MYLK and TKS5 contributes to the ZEB1/miR-200 mediated effect on invasive behavior of cancer cells.

### MYLK is a novel player in invadopodia formation

**Figure 5:**
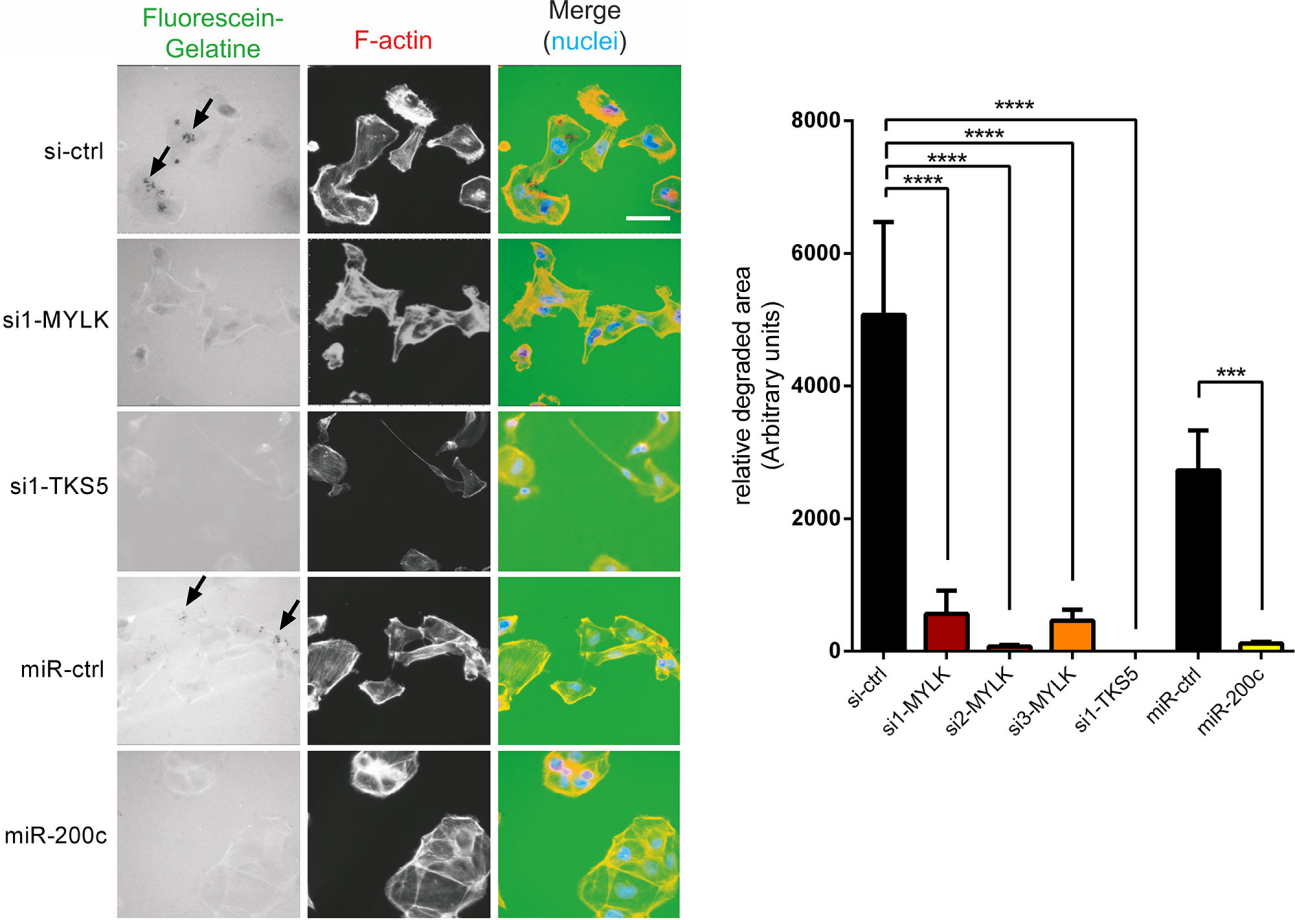
MYLK and TKS5 are essential for the establishment of invadopodia MDA-MB-231, pre-treated with indicated siRNAs or miRNAs, were plated on fluorescein-gelatin coated coverslips. After 24 h cells were fixed, stained and visualized. Representative images are shown. Scale bar, 20 μm. Arrows indicate regions of matrix degradation. 10 random fields of view from three independent experiments were quantified (right) showing significantly reduced gelatin degradation upon transfection of si-MYLK and si-TKS5 to a similar extent as upon overexpression of miR-200c.

Since both of our candidate targets have a clear effect on cell invasion and TKS5 has been well established as a key component of invadopodia [37, 38], we wanted to clarify whether MYLK has any potential role in invadopodia formation as well. Degradation of fluorescently labeled gelatin matrix has been used as a standard method for microscopic evaluation of invadopodia activity [37]. We used this method to analyze the effects of a MYLK knockdown in invasive MDA-MB-231 cells, which revealed a strong reduction of gelatin matrix degradation capacity (Figure 5). Since matrix degradation is the last step in invadopodium formation [39], this directly corresponds to reduced maturation and functionality. Ectopic expression of miR-200c led to a similar effect of reduced invadopodia formation. In agreement with previous findings [37, 38], we observed that knockdown of invadopodia marker TKS5 resulted in a complete loss of gelatin degradation capacity (Figure 5). To further confirm a function of MYLK in invadopodia formation, we used a second assay, the colocalization of F-actin and Cortactin, to directly visualize existing invadopodia [37, 40, 41]. MYLK knockdown led to reduced numbers of detectable invadopodia (Supplementary Figure S5). A similar effect was observed after miR-200c overexpression. In conclusion, these two independent assays demonstrate that MYLK expression is essential for proper invadopodia formation.

### MYLK colocalizes with Cortactin in invadopodia

**Figure 6:**
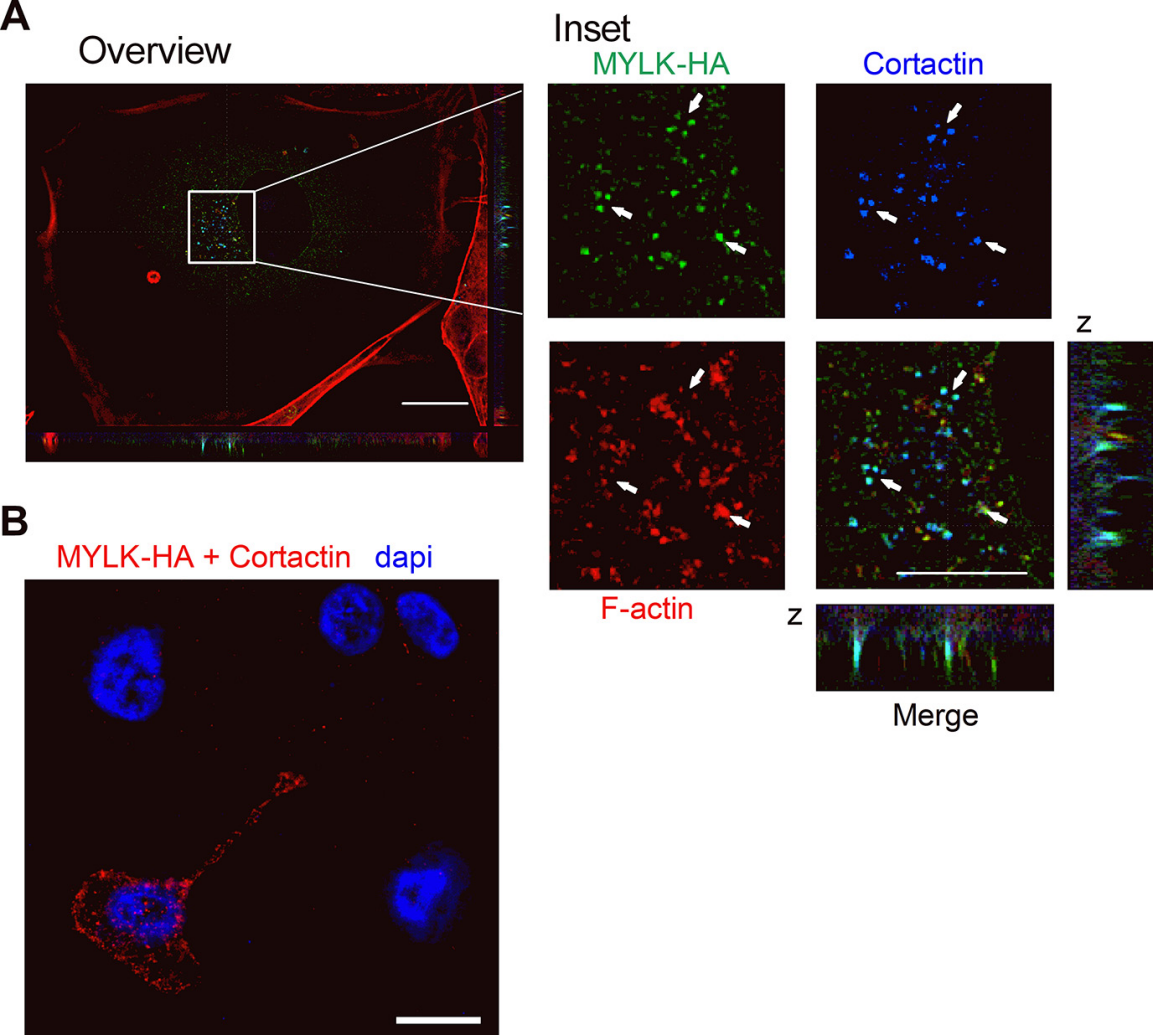
Analysis of MYLK in invadopodia formation **A.** Immunofluorescence showing HA-tagged recombinant MYLK, Cortactin and F-actin (stained with Rhodamine phalloidin) in MDA-MB-231 cells on gelatin-coated coverslips. The three proteins co-localize in point shaped structures that are also obvious in the z axis (merge). Scale bar overview, 20 μm; inset, 10 μm. Arrows indicate points of co-localization. **B.** A representative image of an *in situ* proximity ligation assay (PLA) shows sites of interaction between MYLK-HA and Cortactin in a transfected MDA-MB231 cell indicated by red fluorescent spots. In the untransfected neighboring cells, no signal was observed. Empty vector transfected cells or incubation without antibody were used as negative controls (not shown). Scale bar, 10 μm.

To further understand molecular mechanisms connecting MYLK and invadopodia formation, we first investigated whether its most prominent function, the myosin light chain kinase activity plays a role in this context. MYLK phosphorylates MLC2 at Thr18 and Ser19 [30]. Surprisingly, we observed that knockdown of MYLK as well as overexpression of miR-200c reduced MYLK protein levels, but led to an increase in pMLC2 with no change in total MLC2 levels. Accordingly, when looking at a second substrate, the tyrosine kinase Pyk2, which is activated by MYLK via phosphorylation at Tyr402 [42], neither MYLK knockdown, nor miR-200c overexpression induced a reduction of pPYK2 (Supplementary Figure S6A, S6B). The fact that the phosphorylation status of MYLK substrates was not reduced after MYLK knockdown indicates an additional function, crucial for invadopodia formation and cell invasion. In addition to its kinase activity, the high molecular weight form of MYLK has been shown to interact with Cortactin [31, 43, 44], a major player in invadopodia formation [32]. Therefore, we investigated a putative co-localization of MYLK and Cortactin in invadopodia. Since we could not specifically detect endogenous MYLK by immunofluorescence, we used exogenous expression of an HA-tagged MYLK that was verified in HEK-293T cells (Supplementary Figure S6C). After transient transfection of MYLK-HA in MDA-MB-231 cells plated on gelatin matrix, we observed a clear co-localization of MYLK-HA and Cortactin at the sites of invadopodia (Figure 6A). We confirmed the interaction of both proteins using an *in situ* proximity ligation assay (PLA) (Figure 6B). These data suggest that MYLK could be crucial for invadopodia formation through interaction with Cortactin.

## DISCUSSION

In this work, we could further clarify the detailed functions of the ZEB1/miR-200 feedback loop in tumor invasion by identifying MYLK and TKS5 as two novel miR-200 target genes, that are both necessary for invadopodia formation and invasion of breast cancer cells.

Via *in silico* analysis, we found 17 genes potentially regulated by the ZEB1/miR-200 feedback loop, of which 9 were already described as either direct miR-200 targets (MSN, FN1, MARCKS, FGD1, PPM1F, LOX and KDR [19, 20, 22–24, 26]) or to be inversely correlated with miR-200 expression (QKI, PAG1 [21, 25]), proving the quality of our screen. Additionally, 12 of these 17 genes were already described to be involved in invasion and metastasis [19, 20, 26, 37, 45–51]. Of the 8 remaining novel miR-200 target candidates, we decided to focus on those that showed a significant downregulation after miR-200c overexpression, namely MYLK, TKS5 and WIPF1. Very recently, MYLK, MSN and WIPF1 were also identified as putative miR-200 target genes using a completely different approach. Bracken and colleagues screened for miR-200 interacting proteins using the Ago-HITS-Clip (Argonaute High Throughput Sequencing after Cross-Linked Immunoprecipitation) technology [27]. Since in this publication, WIPF1 was followed up as a miR-200 target, we focused on TKS5 and MYLK for further characterization.

Tyrosine kinase substrate with five SH3 domains (TKS5/SH3PXD2A) is known to act in invadopodia formation as a SRC dependent scaffolding protein which recruits different effector proteins, including Cortactin and ADAM metalloproteases to initiate actin polymerization and extracellular matrix degradation [28, 29]. TKS5 knockdown abolishes invadopodia formation and extracellular matrix degradation in human breast cancer and melanoma cells [37] and inhibits the formation of lung metastases by SRC-transformed mouse fibroblasts and Ras-transformed human mammary epithelial cells after tail vein injection or subcutaneous transplantation [14, 52].

Myosin light chain kinase (MYLK), is a protein kinase, whose main known function is the phosphorylation of myosin light chain (MLC2) at Thr18 and Ser19, that is a prerequisite for the formation of contractile actomyosin-filaments [30]. The short isoform of MYLK is mainly expressed in smooth muscle tissue and contains a central catalytic domain, a CaM-binding element, three Ig-modules, a fibronectin-module and three N-terminal F-actin binding motives [53, 54]. The long isoform that dominates in non-muscle tissue harbors additional six Ig-modules and two actin binding motifs, mediating a higher affinity to actin filaments [55–57]. This non-muscle specific isoform was shown to directly interact with Cortactin [31] that is also a SRC kinase dependent crucial factor for invadopodia formation [31, 32] and plays a role in aggressive cancers [58].

We could show that MYLK and TKS5 are indeed direct mir-200c target genes, which expression patterns correlate positively with that of ZEB1 in different datasets of cancer cell lines. Pointing out to an involvement in EMT, both genes also cluster in the mesenchymal group of the NCI60 cell lines [6], whereas miR-200c is expressed mainly in the epithelial group. Indicating clinical relevance, MYLK and TKS5 levels also correlate positively with ZEB1-, but negatively with E-cadherin and miR-200-levels in human breast cancer samples.

The biological relevance is further supported by our result, that siRNA mediated knockdown of either gene reduced the invasive potential of breast cancer cells in matrigel, mediating at least in part the miR-200c effect. These findings are in line with previous publications showing that TKS5 [37, 59, 60] as well as MYLK [50, 61] are important for the invasion of cancer cells. In contrast to miR-200c overexpression, we found no significant reduction of migration capacity upon knockdown of TKS5 or MYLK. For TKS5, this is in agreement with its published function as a scaffolding protein in the formation of invadopodia, which are cellular protrusions that degrade the extracellular matrix in invasive cancer cells [62]. For MYLK, its role in cell migration remains controversial. In some publications MYLK was shown to be essential for invasion as well as for migration via MLC2 phosphorylation dependent Myosin II activation in cancer cells [61, 63]. But these and most other studies, regarding the MLC2 related MYLK function are controversial, since they were done using MYLK kinase inhibitors that were shown to be non-specific [64, 65]. Other studies even observe the opposite effect. Simpson and colleagues found accelerated migratory behavior after MYLK siRNA treatment in an siRNA screen in MCF10 epithelial cells [66]. Chen et al. [67] also describe a higher migratory potential of MYLK−/− smooth muscle cells in comparison to wt, that is not dependent on the kinase function. Since we expected, if any, an inhibitory effect after MYLK knockdown and found no significant changes, we focused on the invasion capacity of our cells. The anti-migratory effect of miR-200c itself could be mediated by the inhibition of other motility-linked target genes like MSN, FN1 [19], FHOD or PPM1F [26].

According to the similar effects of MYLK- and TKS5-depletion on invasion capabilities, we found that MYLK also plays a role in invadopodia formation. Comparable to the impact of miR-200 overexpression, knockdown of MYLK dramatically diminished invadopodia assembly and functionality. Therefore, we identified MYLK as a novel player in invadopodia formation that mediates this anti-invasive miR-200 effect together with TKS5 and WIPF1 [27].

The main published function of MYLK is to phosphorylate and thereby activate MLC2, which is important for cell morphology, contraction, motility and other membrane events like apoptotic blebbing [30]. When analyzing the MLC2 phosphorylation status upon either direct siRNA mediated knockdown or suppression via miR-200c overexpression, we detected an increase of phospho-MLC2, instead of the expected decrease, whereas total MLC2 amounts remained unaffected. Confirming these results, analysis of another MYLK substrate, PYK2 [42], revealed no reduction in phosphorylation after MYLK depletion. Since also other kinases like ROCK, CRIK, DAPK3 or MRCK are able to phosphorylate MLC2 [68], one or several of these might compensate for the loss of MYLK. The intact phosphorylation status of MLC2 is in accordance with the unaffected migratory capability of the cells. However, MYLK still has an important function in invasion and invadopodia formation. Previous publications identified the long isoform of MYLK in a complex with Cortactin and SRC [44], and confirmed the direct interaction of MYLK and Cortactin and the phosphorylation of both via SRC kinase [31]. Accordingly, we detected MYLK together with Cortactin in invadopodia. Since the SRC-mediated activation of Cortactin is essential for invadopodia assembly [39], this interaction might be the key to MYLK function in invadopodia formation.

In summary, we here identified two direct miR-200c target genes, TKS5 and MYLK that are both necessary for invasion and invadopodia formation of breast cancer cells. Whereas TKS5 is a well-known factor in invadopodia formation, MYLK is a novel player that might function through Cortactin interaction. TKS5 and MYLK represent two effectors/mediators of the invasive behavior of cancer cells that are regulated by the ZEB1/miR-200 feedback loop.

## MATERIALS AND METHODS

### Cell lines and cell culture

Human breast cancer cells lines MDA-MB-231, BT-549, HS578T, MCF7, T47D as well as human embryonic kidney 293T cells were obtained from American Type Culture Collection (ATCC). The MDA-MB-231 stable knockdown clones for ZEB1 (shZEB1) and the control clone (shCtrl) were described earlier [9]. All cell lines were cultured in Dulbecco's Modified Eagle Medium (DMEM; Life Technologies) containing 10% fetal bovine serum (FBS; Gibco) in a humidified incubator at 37°C and 5% CO_2_.

### Transfection and luciferase reporter gene assays

Plasmid transfections were carried out using Lipofectamine LTX with Plus reagent (Life Technologies) as recommended by the manufacturer. siRNA and miRNA transfections were carried out using Lipofectamine RNAiMAX transfection reagent (Life Technologies). siRNAs, miRNAs, antagomiRs were used to a final concentration of 20, 15 and 100 nM respectively. Equal amounts of antagomiRs for miR-200b, -c and -429 were combined for effective inhibition. 2 μg of expression vector for MYLK (pCI-neo-MYLK-HA) were transfected per six-well. Transfections for gene expression and immunoblot assays were harvested after 72 h of treatment. For luciferase reporter assays, cells were transfected with miRNAs or antagomiRs on day 1. On day 3, reporter plasmids were cotransfected with Renilla luciferase construct (pRL-TK, Promega) and incubated for an additional 24 h. Luciferase activity was measured using the Dual-Luciferase Reporter Assay System (Promega).

### RNA isolation and quantitative RT-PCR

Total RNA was extracted using the RNeasy Plus Mini Kit (Qiagen) according to the manufacturer's instructions for inclusion of miRNAs. 1 μg of RNA was reversely transcribed using the RevertAid First Strand cDNA Synthesis Kit (Thermo Scientific). cDNA for miRNAs was synthesized using Universal cDNA Synthesis Kit II (Exiqon) following the manufacturer's protocol. mRNA and miRNA expression levels were measured using Power SYBR Green PCR Master Mix (Applied Biosystems) and miRCURY LNA Universal RT microRNA PCR-Kit (Exiqon), respectively. Expression values were measured on a Roche LightCycler 480 and normalized to β-Actin expression for mRNA and to miR-16 for miRNA. Relative expression for all samples were calculated using the Pfaffl method [69]

### Western blotting

Immunoblots were performed as described previously [9]. 50–80 μg protein lysate were separated by SDS-PAGE, transferred to nitrocellulose membranes and incubated with the following primary antibodies: anti-α-Tubulin (Sigma, T6199; 1:5000), anti-β-Actin (Sigma, A5441; 1:5000), anti-HA (Roche, 3F10; 1:1000), anti-MLC2 (Cell Signaling Technology, 3672; 1:1000), anti-MYLK (Abcam, EP1458Y; 1:1000), anti-pMLC2 (Cell Signaling Technology, 3674; 1:1000), anti-pPYK2 (Cell Signaling Technology, 3291; 1:1000), anti-TKS5 (Santa Cruz, sc-30122; 1:1000) and anti-ZEB1 (Sigma Prestige, HPA027524; 1:5000).

### Immunofluorescence

Cells were fixed with 4% formaldehyde or ice-cold methanol and blocked with PBS/2% normal goat serum. Fixed cells were incubated with the primary antibodies rat anti-HA (Roche, 3F10; 1:300), rabbit anti-HA (Sigma-Aldrich, H6908, 1:300), mouse anti-Cortactin (MerckMillipore, 4F11; 1:500), mouse anti-E-cadherin (BD Biosciences, 610182, 1:1000) and rabbit anti-TKS5 (Santa Cruz, sc-30122, 1:100) as indicated in the text at 4°C overnight, followed by Alexa Fluor_ 488-conjugated donkey anti-rat IgG (1:500, A21208; Life Technologies), Alexa Fluor_ 350-conjugated goat anti-mouse IgG (1:500, A11045; Life Technologies), Alexa Fluor_ 594-conjugated goat anti-mouse IgG (1:500, A21135; Life Technologies) or Alexa Fluor_ 488-conjugated goat anti-rabbit IgG (1:500, A-11029; Life Technologies) for 1 h at room temperature and counterstained with DAPI (Molecular Probes).

F-actin was stained by Alexa fluor 488 phalloidin or Rhodamine phalloidin (1:100, A12379, R415, Life Technologies).

### Plasmid construction

Plasmid expressing C-terminally 2X-HA-tagged MYLK (pCI-neo-MYLK-HA) was generated by cDNA amplification and cloning of the full-length coding sequence of human MYLK into the mammalian expression vector (pCI-neo; Promega) using primers containing *Xho*I and *Not*I restriction sites. For cloning of human MYLK 3′UTR and human TKS5 3′UTR reporters, nucleotides + 6027 to + 7852 and + 5150 to + 5650 respectively were amplified from cDNA and cloned downstream of *Renilla* luciferase open reading frame in the pRL-TK (Promega). For mutant reporters, four basepairs (indicated in Supplementary Table S1) were deleted from the miRNA seed binding sequences by site directed mutagenesis (QuikChange, Agilent Technologies).

### Migration and invasion assays

To assess cell migration in a wounding assay, cells were transfected with siRNAs, miRNAs and controls. After reaching confluence, cells were scratched with a pipette tip and the migration potential was observed at 4, 8 and 24 h. Invasive capacity was analyzed using a *Cultrex BME Cell Invasion Assay* (Trevigen) according to the manufacturer's instructions. In brief, after serum starvation for 24 h, 4 × 10^4^ transfected cells were seeded in the upper chamber of a transwell plate on the matrigel coated membrane. 24 h later, the amount of cells that had invaded into the 10% FBS containing bottom chamber was analyzed.

For *xCELLigence* Real-Time Cell Analysis (RTCA) migration assays, cells were plated into the upper chamber of RTCA Cim-16 plates (Roche) in a serum free medium. For invasion assays, cells were plated into the upper chamber precoated with 1:40 diluted Matrigel (BD Biosciences). In both assays medium with 10% FBS was used as a chemo attractant in the lower chamber. Electrical impedance generated by cells that migrated or invaded and settled to the lower chamber was measured in a real-time setting using the xCELLigence RTCA DP instrument (Roche Diagnostics) in a humidified incubator at 37°C and 5% CO_2_ and analyzed using the RTCA Software 1.2 (Roche Diagnostics). The slope of the graphs between 5–20 h for migration and between 9–30 h for invasion was used for quantification. All values were normalized to its respective control.

### Gelatin degradation assay

Fluorescein-gelatin coating of coverslips was performed using the QCM Gelatin Invadopodia assay kit (Millipore) according to manufacturer's instructions. Cells were seeded on fluorescein-gelatin coated coverslips 24 h post-transfection and incubated for another 24 h. Following fixation with 4% paraformaldehyde (PFA), F-actin was labeled using rhodamine-phalloidin (Life technologies; 1:50) and nuclei were stained with Hoechst dye (Molecular Probes). The gelatin degradation area was measured according to the manufacturer's instructions using the ImageJ software and normalized to the cell number (determined by Hoechst-stained nuclei).

### F-actin/Cortactin colocalization assay

Coverslips were prepared as above mentioned with 0.2% porcine gelatin/PBS (Sigma-Aldrich) and cells were seeded 24 h after transfection. After another 24 h, the cells were fixed and stained for Cortactin (EMD Millipore; 1:1000) and Alexa Flour 488-conjugated phalloidin (Life Technologies; 1:50). For the analysis, 8 fields of view per coverslip from three biological replicates were imaged using a confocal microscope and analyzed for colocalization using the ImageJ software.

### Proximity ligation assay (PLA)

Proximity ligation assay for detection and localization of protein-protein interactions *in situ* was done with the Duolink in situ reagent kit. Transfected cells were seeded on gelatin coated coverslips and fixation, permeabilization, blocking and staining with the primary antibodies was performed as for immunofluorescence. Samples were then washed once with washing buffer (0.02% Tween20/PBS) and anti-rabbit plus and anti-mouse minus PLA probes were incubated in 1.5% BSA/PBS blocking solution for 1 h at 37°C in a pre-heated humidity chamber. Ligation, Amplification and washing were done according to the manufacturer's instructions.

### Correlation analysis

The expression datasets of 101 breast cancer patients' samples and of the CCLE panel of cancer cell lines were obtained from the NCBI-GEO database (GEO accession no. GSE19783 [35] and GSE36133 [34]). For analysis of genes and miRNA expression in the NCI60 panel, normalized expression levels were obtained from the CellMiner database 1.5.1 (http://discover.nci.nih.gov/cellminer/). Expression data for each cell line in the NCI60 panel were further classified into epithelial, undefined and mesenchymal types based on EMT marker expression [6]. All heat maps were generated using the GENE-E software (Broad Institute).

### Statistical analysis

All experiments were performed in at least three biological replicates. All statistical and correlation analyses were performed using the GraphPad Prism software (GraphPad Software, Inc.). All intervals were expressed as mean ± SEM. Comparison between two groups (qRT-PCR, reporter assays) was performed using one- (Figure 1D) or two-tailed Student's *t*-test. Comparison among three or more groups was performed using one-way analysis of variance (ANOVA) with Bonferroni post-hoc *t*-test. Statistical significance in every figure was represented as follows: *P ≤ 0.05; **P ≤ 0.01; ***P ≤ 0.001; ****P ≤ 0.0001; n.s. not- significant.

All siRNA, miRNA, antagomiR sequences and primers used for cloning or qRT-PCR are listed in the Supplementary Table S1.

## SUPPLEMENTARY FIGURES AND TABLE


