# Supplementary material for: The ZEB1/miR-200c feedback loop regulates invasion via actin interacting proteins MYLK and TKS5
Source: Oncotarget. 2015 Aug 20;6(29):27083–96. doi: 10.18632/oncotarget.4807 (PMC4694975; doi:10.18632/oncotarget.4807)
Supplement: Supplementary file 1 [file oncotarget-06-27083-s001.pdf]

SUPPLEMENTARY FIGURES AND TABLE

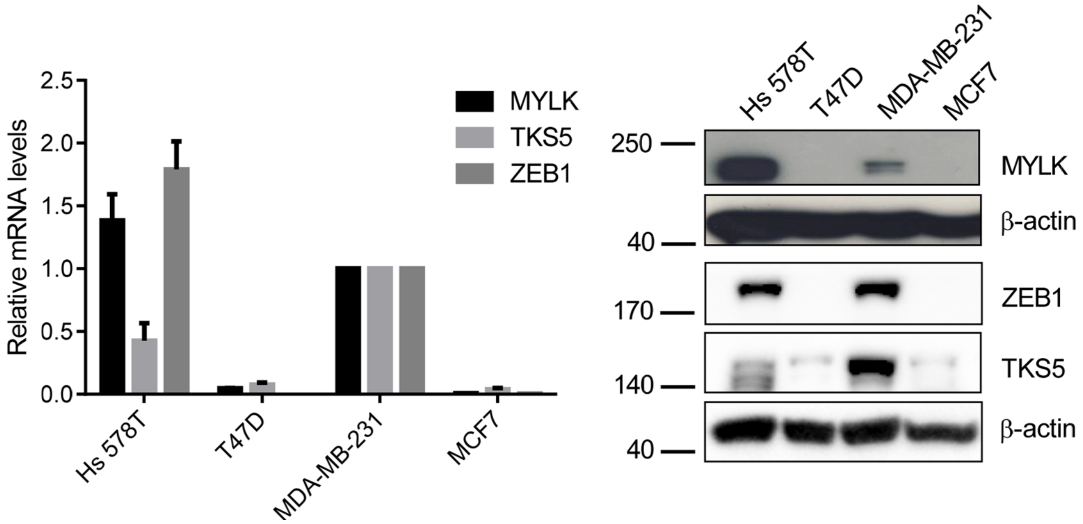

Supplementary Figure S1: MYLK and TKS5 are enriched in mesenchymal basal type breast cancer cell lines. Quantitative RT-PCR (left) and immunoblots (right) showing expression levels of MYLK, TKS5, ZEB1 in epithelial (T47D and MCF7) and mesenchymal (Hs 578T and MDA-MB-231) breast cancer cell lines. ACTB was used for normalization in qPCRs and β-Actin as loading control in immunoblots.

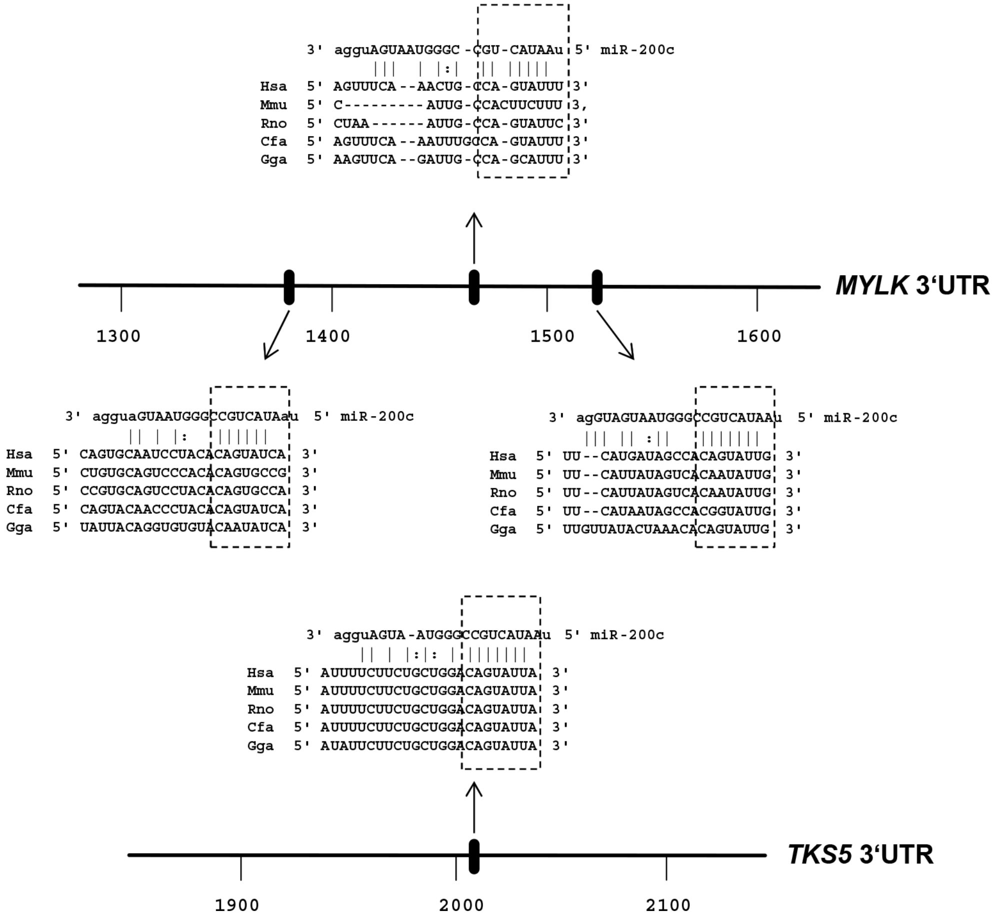

Supplementary Figure S2: MYLK and TKS5 mRNAs contain miR-200c target sequences. Schematic representation of putative miR-200c binding sites in the 3′UTRs of MYLK and TKS5 showing high interspecies conservation of seed matching sequences (dotted box).

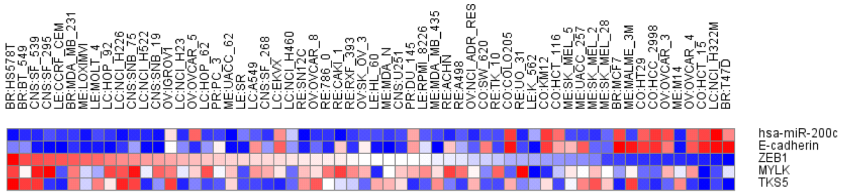

NCI60 panel	*MYLK* vs. *miR-200c*	*TKS5* vs. *miR-200c*	*ZEB1* vs. *miR-200c*	*MYLK* vs. *ZEB1*	*TKS5* vs. *ZEB1*
r	- 0.4740	- 0.4091	- 0.5729	0.4073	0.2777
P-value	0.0001	0.0012	0.0001	0.0012	0.0317

Supplementary Figure S3: Expression correlation of *MYLK* and *TKS5* in NCI60 panel. Heat map showing expression levels of *miR-200c, E-cadherin, ZEB1, MYLK and TKS5* in the NCI60 tumor cell line panel obtained from publicly available CellMiner database. Pearson correlation coefficients (*r*) and *P*-values computed are shown below the map.

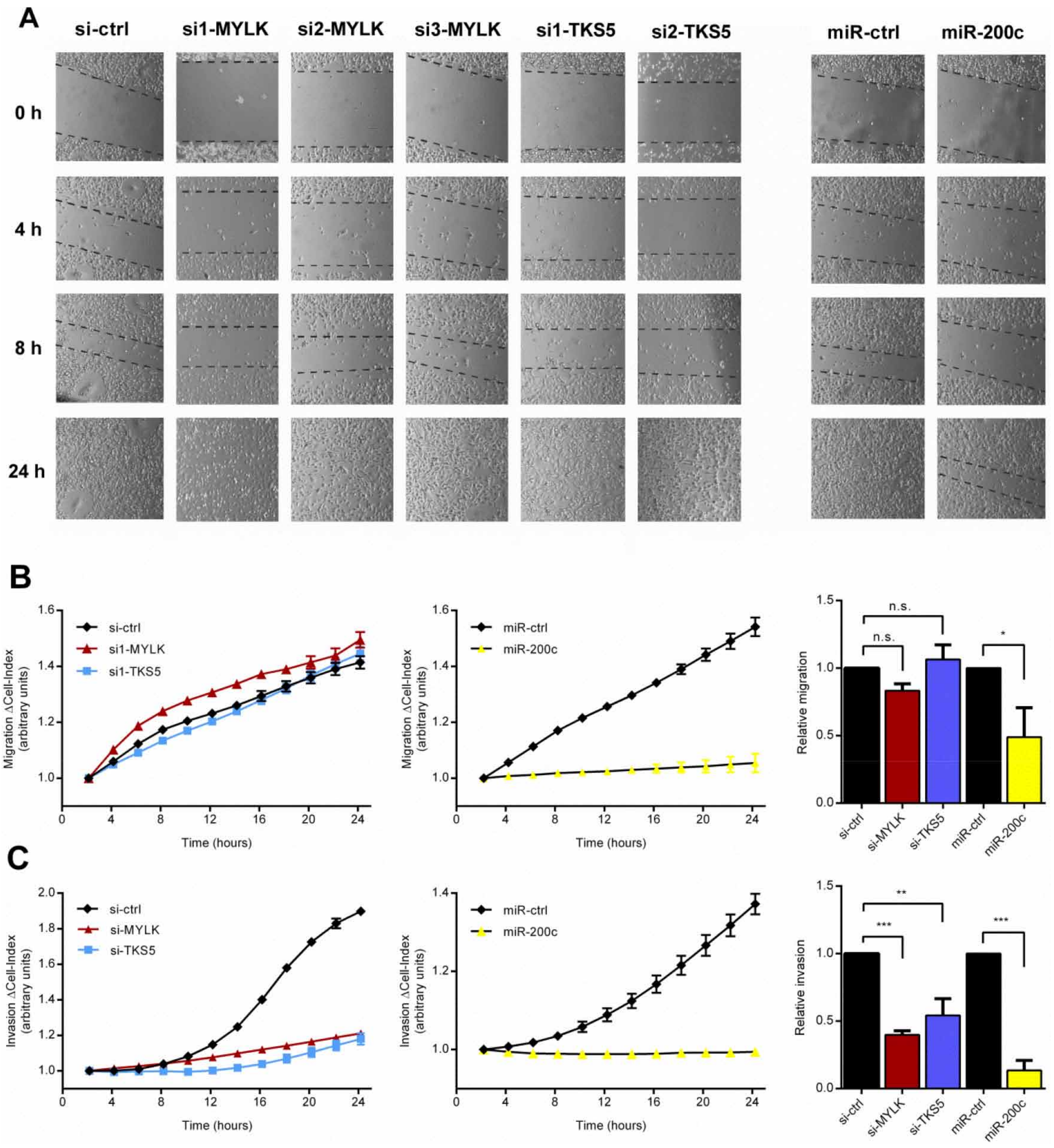

Supplementary Figure S4: Knockdown of MYLK or TKS5 does not influence migration but inhibits cancer cell invasion. A. Representative images of wounding assays using MDA MB 231 cells transfected with either siRNA against MYLK, TKS5 or si-ctrl, or with miR-200c or miR-ctrl. **B.** Representative graphs of RTCA migration assays for MDA-MB-231, transfected with si1-MYLK, si1-TKS5, si-ctrl, miR-200c or miR-ctrl. Quantification of three biological replicates (right) shows a significant reduction in migration relative to control cells only after miR-200c overexpression, but not after si-MYLK or si-TKS5 treatment. **C.** Representative graphs of RTCA invasion assays for MDA-MB-231, transfected with si-MYLK, si-TKS5 or miR-200c. Quantification of three biological replicates (right) shows a significant reduction in invasion relative to control cells after si1-MYLK or si1-TKS5 treatment as well as after miR-200c overexpression.

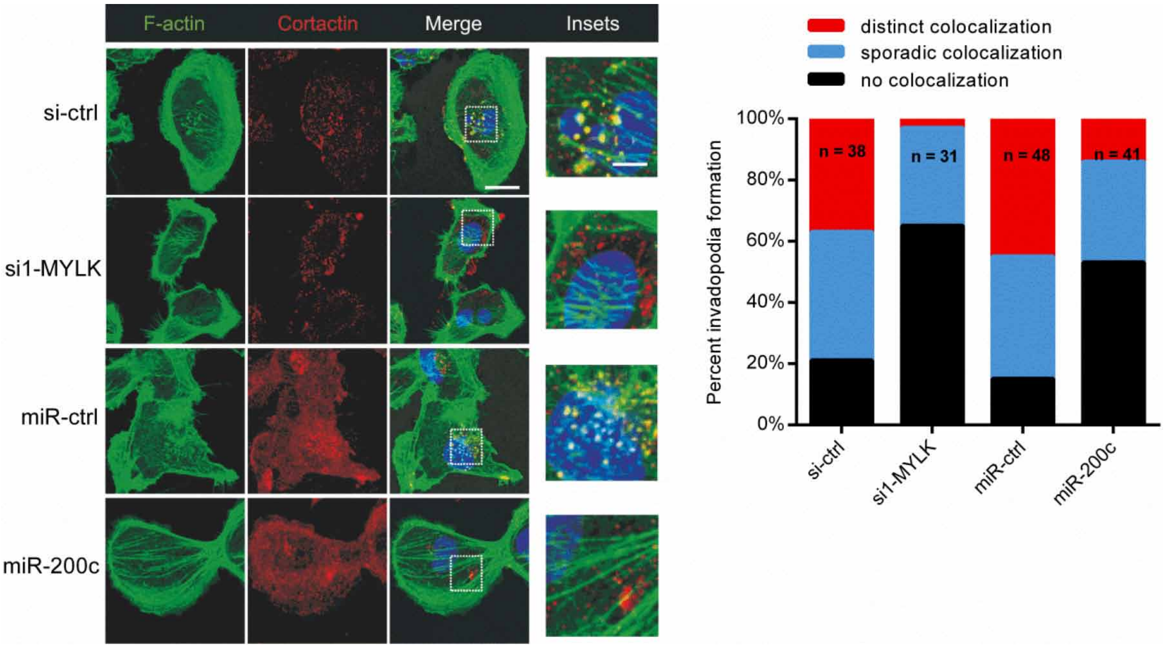

Supplementary Figure S5: MYLK knockdown reduces the establishment of invadopodia. MDA-MB-231, pre-treated with indicated siRNAs or miRNAs, were plated on gelatin-coated coverslips. After 24 h cells were fixed, stained for F-actin and Cortactin, and imaged using a confocal microscope. Scale bar, 10 µm. Insets show a higher magnification of the observed colocalization as depicted with scale bar, 30 µm. Individual analyses of indicated cell numbers (from three independent experiments) were analyzed (right) for the presence of distinct, sporadic or absence of colocalization. A similar reduction of F-actin/Cortactin colocalization was observed after either si-MYLK or miR-200c transfection.

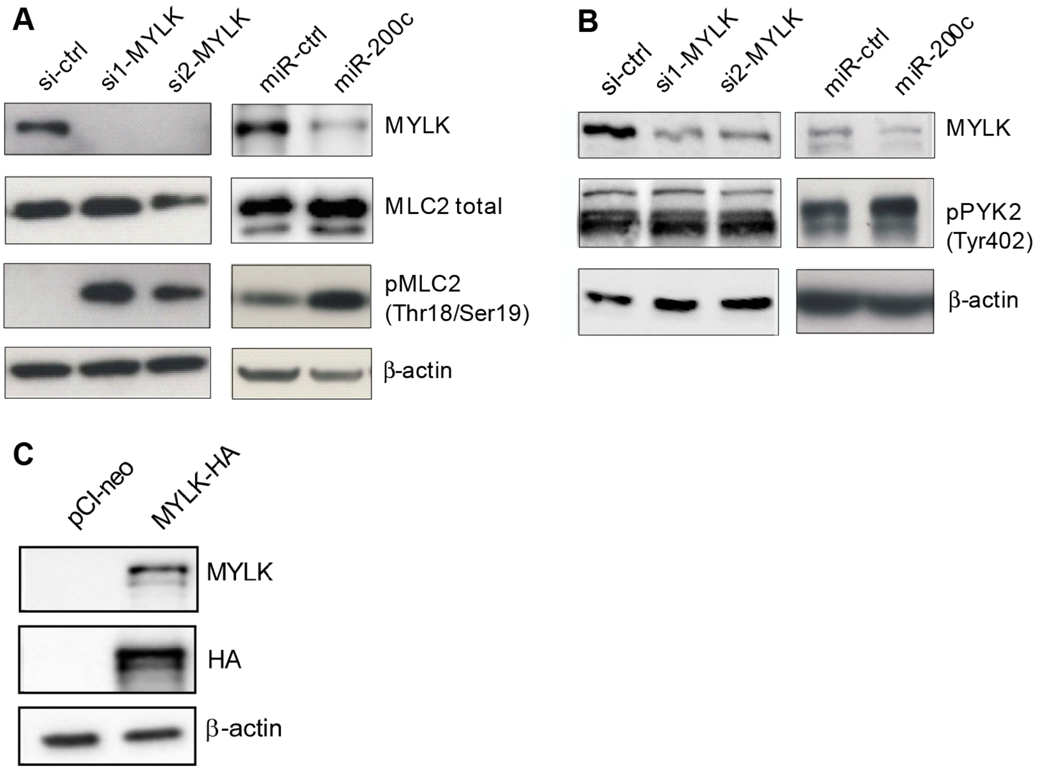

Supplementary Figure S6: Recombinant expression of full-length MYLK. A, B. Immunoblots of MDA-MB-231, showing that the phosphorylation status of the main MYLK target MLC2 is elevated relative to the respective control after si-MYLK or miR-200c treatment (pMLC2 (Thr18/Ser19) and MLC2total) (A), whereas the phosphorylation status of the MYLK substrate PYK2 (pPYK2 (Tyr402)) does not change (B) As expected, MYLK levels were reduced after miR-200c overexpression or MYLK siRNA treatment. β-Actin was used as loading control. **C.** Immunoblot image showing validation of transient overexpression of full-length MYLK-cDNA in HEK-293T cells, also detected using HA antibody. pCI-neo represents the empty vector, β-Actin was used as loading control.

Supplementary Table S1: Oligonucleotides used in this study

**miRNA sequences**
hsa-miR-200c, 5‘-UAAUACUGCCGGGUAAUGAUGGA-3‘ (AM17101)
Pre-miR Negative Control (AM17111)

**antagomiR sequences**
hsa-anti-miR-200b, 5′-UCAUCAUUACCAGGCAGUAUUA-3′ ;
hsa-anti-miR-200c, 5′-UCCAUCAUUACCCGGCAGUAUUA-3′;
hsa-anti-miR-429, 5′-ACGGUUUUACCAGACAGUAUUA-3′

**siRNA sequences**
si1 MYLK, 5′-GCCUCAUGUAAAACCCUAUtt-3′ (Silencer Select s9193);
si2 MYLK, 5′-GGACGGGAACUGCUCUUUAtt-3′ (Silencer Select s9194);
si3 MYLK, 5′- GACCAUUCGCGAUUUAGAAtt-3′ (Silencer Select s9195);
si1 TKS5, 5′- GCUGGUGGUAUAUCAGAUAtt-3′ (Silencer select s18542);

(*Continued*)

si2 TKS5, 5′- GGUCAUUGAUAAGAACUCAtt-3′ (Silencer select s228519);
siRNA Negative Control (Ambion 4390847)

miRNAs and siRNAs were purchased from Life Technologies and antagomiRs were purchased from Dharmacon, Thermo Scientific.

**Primers used for qRT-PCR**	
hsa-ZEB1,	for 5′-AAGAATTCACAGTGGAGAGAAGCCA-3′,
	rev 5′-CGTTTCTTGCAGTTTGGGCATT-3′
hsa-MYLK,	for 5′-CCGCTCAATGCAGAAAAACTA-3′
	rev 5′-GGTTTTACATGAGGCTTTTCCTC-3′
hsa-TKS5,	for 5′-TCGCTTTGTGGGGGAAGATG-3′
	rev 5′-GTCTGGGAGGTGGAGTCAGA-3′
hsa-CORO1C,	for 5′-TTGCCATAATCATAGAGGCAAGT-3′
	rev 5′-TTTGTCAATTCGACCAGTCTTG-3′
hsa-TRIO,	for 5′-AGTCCACCCAGAGCAACG-3′
	rev 5′-CGTGTAATCGTGTGTCACCAA-3′
hsa-WIPF1,	for 5′-GAAACGAAAGCCGGAGTG-3′
	rev 5′-AAAGATCACCTCGGGATGG-3′
hsa-NR3C1,	for 5′-CCTTCTGCGTTCACAAGCTA-3′
	rev 5′-TTCTTTGGAGTCCATCAGTGAAT-3′
hsa-SASH1,	for 5′-AATTGAGGAAGCACTTGCTAGG-3′
	rev 5′-ACCATCTGGCCAGTCAGC-3′
hsa-AMOTL2,	for 5′-AGGCTGCAGAGAGACAATGAG-3′
	rev 5′-CTCAGAGAGCCGCTGGATT-3′

**Primers used for 3′UTR reporter cloning**	
MYLK 3′UTR	for 5′-AACAAAGCCAGAGAAAAGCAGT-3′
	rev 5′-TTGAACAAACAGCATGCACTG-3′
TKS5 3′UTR	for 5′-ACTGGGGGTTGGTGTTTTCT-3′
	rev 5′-GCACTTTGCTCTCTGGGTTT-3′

**Primers used for 3′UTR reporter mutagenesis**
MYLK 3′UTR mut1
for 5′-TTTTGCTGCTAGTTTCAAACTGCCTTTTTCCTTTTGCTTTTAAAATAGT-3′
rev 5′-ACTATTTTAAAAGCAAAAGGAAAAAGGCAGTTTGAAACTAGCAGCAAAA-3′
MYLK 3′UTR mut2
for 5′-GTTACAATATTTTTCATGATAGCCACTTGCCACAGTTTATTATAATAAAGGG-3′
rev 5′-CCCTTTATTATAATAAACTGTGGCAAGTGGCTATCATGAAAAATATTGTAAC-3′

(*Continued*)

MYLK 3′UTR mut3
for 5′-AAAATTACTTCAGTGCAATCCTACACTCAACATTAGAATTTTGATATTAGTC-3′
rev 5′-GACTAATATCAAAATTCTAATGTTGAGTGTAGGATTGCACTGAAGTAATTTT-3′
TKS5 3′UTR mut
for 5′-GAGTTGTAGGACGAGATAAATACAGGTCGTCTTCTTTTATTG-3′
rev 5′-CAATAAAAGAAGACGACCTGTATTTATCTCGTCCTACAACTC-3′

Four nucleotides of the miR-200c binding site were deleted during mutagenesis. The two adjacent basepairs are underlined.

**Primers used for cloning full-length MYLK-cDNA**	
for	5′-ATGGGGGATGTGAAGCTG-3′
rev	CTAGAACTCAGCATAATCTGGGACGTCATAAGGATATCCAGCATAATCTGGCACGTCAT AAGGATACTCTTCTTCCTCTTCCCCTTC-3′

In the reverse primer 2x HA tag sequence shown in bold characters followed by stop codon underlined.
